# (*E*)-3-(1,3-Benzodioxol-5-yl)-2-{[*N*-(2-formylphenyl)-4-methylbenzenesulfon­amido]methyl}prop-2-enenitrile

**DOI:** 10.1107/S1600536812042663

**Published:** 2012-10-20

**Authors:** M. Bakthadoss, A. Devaraj, R. Madhanraj, S. Murugavel

**Affiliations:** a Department of Organic Chemistry, University of Madras, Maraimalai Campus, Chennai 600 025, India; b Department of Chemistry, Pondicherry University, Puducherry 605 014, India; c Department of Physics, Ganadipathy Tulsi’s Jain Engineering College, Kaniyambadi, Vellore 632 102, India; d Department of Physics, Thanthai Periyar Government Institute of Technology, Vellore 632 002, India

## Abstract

In the title compound, C_25_H_20_N_2_O_5_S, the benzodioxole ring system is essentially planar [maximum deviation = 0.021 (2) Å] and forms dihedral angles of 85.2 (1) and 74.2 (1)°, respectively, with the formyl benzene and sulfonyl-bound benzene rings. In the crystal, C—H⋯O hydrogen bonds generate *C*(8) chains along [100] and *R_3_^3^*(19) ring motifs. In addition, a weak π–π inter­action [centroid–centroid distance = 3.937 (3) Å] is also observed.

## Related literature

For background to the pharmacological uses of sulfonamides, see: Korolkovas (1988 ▶); Mandell & Sande (1992 ▶). For benzodioxole derivatives, see: Ullrich *et al.* (2004 ▶); Gates & Gillon (1974 ▶); Arndt & Franke (1977 ▶); Joshi *et al.* (2005 ▶); Jae *et al.* (2001 ▶); Leite *et al.* (2004 ▶). For related structures, see: Madhanraj *et al.* (2011 ▶); Aziz-ur-Rehman *et al.* (2010 ▶). For hydrogen-bond motifs, see: Bernstein *et al.* (1995 ▶). For the Thrope–Ingold effect, see: Bassindale (1984 ▶).
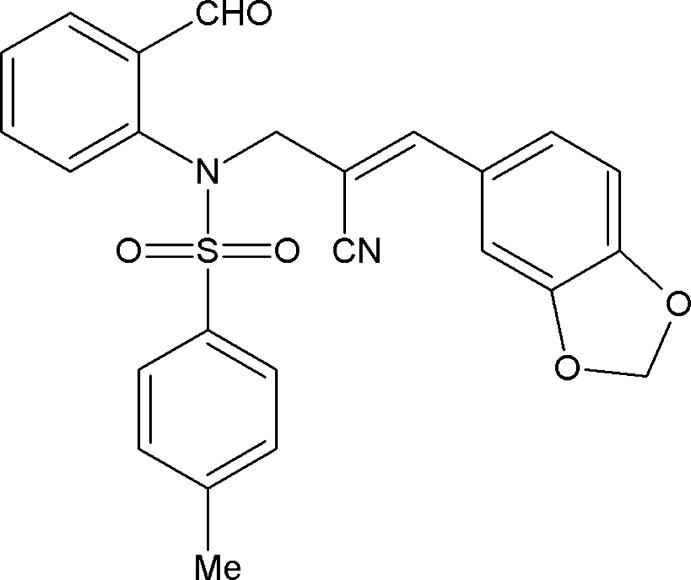


## Experimental

### 

#### Crystal data

C_25_H_20_N_2_O_5_S
*M* *_r_* = 460.49
Monoclinic, 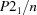

*a* = 8.921 (5) Å
*b* = 10.235 (4) Å
*c* = 25.256 (3) Å
β = 93.380 (4)°
*V* = 2302.0 (16) Å^3^
*Z* = 4
Mo *K*α radiation
μ = 0.18 mm^−1^
*T* = 293 K
0.23 × 0.21 × 0.16 mm

#### Data collection

Bruker APEXII CCD diffractometer
Absorption correction: multi-scan (*SADABS*; Sheldrick, 1996 ▶) *T*_min_ = 0.959, *T*_max_ = 0.972
26810 measured reflections
6451 independent reflections
3582 reflections with *I* > 2σ(*I*)
*R*_int_ = 0.035

#### Refinement

*R*[*F* ^2^ > 2σ(*F* ^2^)] = 0.050
*wR*(*F* ^2^) = 0.148
*S* = 1.01
6451 reflections
299 parameters
H-atom parameters constrained
Δρ_max_ = 0.24 e Å^−3^
Δρ_min_ = −0.28 e Å^−3^

### 

Data collection: *APEX2* (Bruker, 2004 ▶); cell refinement: *APEX2* and *SAINT* (Bruker, 2004 ▶); data reduction: *SAINT* and *XPREP* (Bruker, 2004 ▶); program(s) used to solve structure: *SHELXS97* (Sheldrick, 2008 ▶); program(s) used to refine structure: *SHELXL97* (Sheldrick, 2008 ▶); molecular graphics: *ORTEP-3* (Farrugia (1997 ▶); software used to prepare material for publication: *SHELXL97* and *PLATON* (Spek, 2009 ▶).

## Supplementary Material

Crystal structure: contains datablock(s) global, I. DOI: 10.1107/S1600536812042663/bt6843sup1.cif

Structure factors: contains datablock(s) I. DOI: 10.1107/S1600536812042663/bt6843Isup2.hkl

Supplementary material file. DOI: 10.1107/S1600536812042663/bt6843Isup3.cml

Additional supplementary materials:  crystallographic information; 3D view; checkCIF report

## Figures and Tables

**Table 1:** Hydrogen-bond geometry (Å, °)

*D*—H⋯*A*	*D*—H	H⋯*A*	*D*⋯*A*	*D*—H⋯*A*
C15—H15*B*⋯O2^i^	0.97	2.42	3.282 (3)	148
C23—H23⋯O1^ii^	0.93	2.41	3.114 (3)	132
C4—H4⋯O3^iii^	0.93	2.59	3.195 (3)	124

Symmetry codes: (i) 
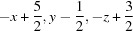
; (ii) 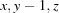; (iii) 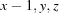.

**Fig. 1.**
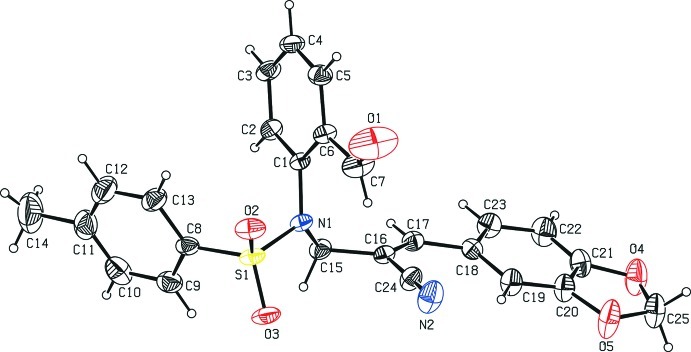
The molecular structure of the title compound with the atom numbering scheme. Displacement ellipsoids are drawn at the 20% probability level. H atoms are presented as a small cycles of arbitrary radius.

**Fig. 2.**
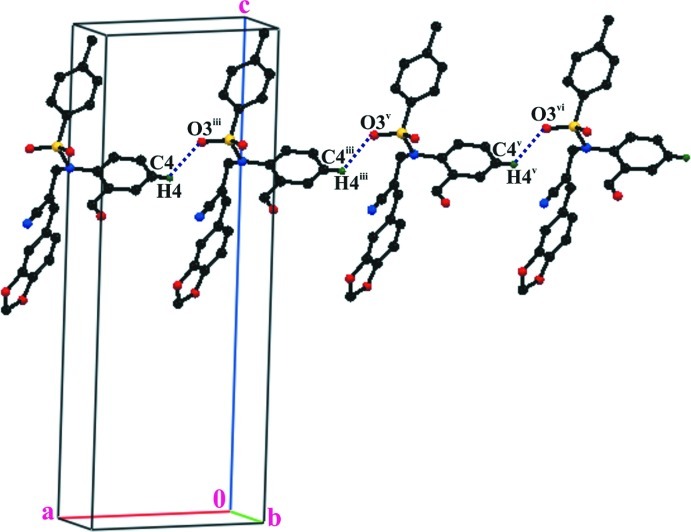
Part of the crystal structure of (I) showing C—H···O hydrogen bonds (dotted lines), with the formation of C(8) chains along [100]. [Symmetry codes: (iii)-1+x, y, z; (v)-2+x, y, z; (vi)-3+x, y, z].

**Fig. 3.**
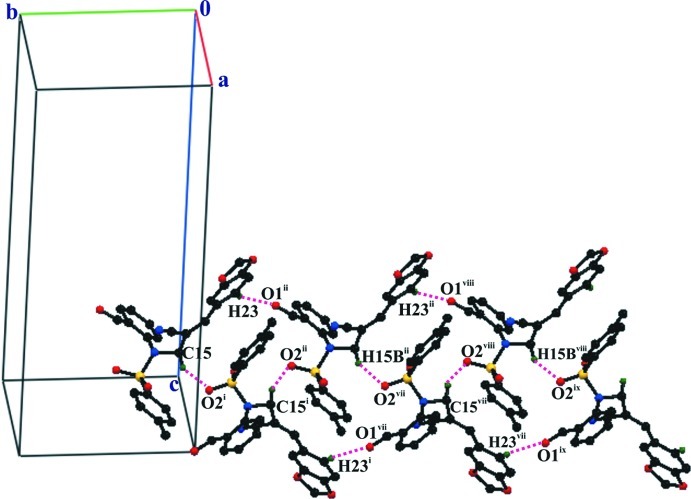
Part of the crystal structure of (I) showing C—H···O hydrogen bonds (dotted lines), with the formation of R33(19) ring motifs along [010] [Symmetry codes: (i)5/2-x, -1/2+y, 3/2-z; (ii)x, -1+y, z; (vii)5/2-x, -3/2+y, 3/2-z; (viii)x, -2+y, z; (ix)5/2-x, -5/2+y, 3/2-z].

**Fig. 4.**
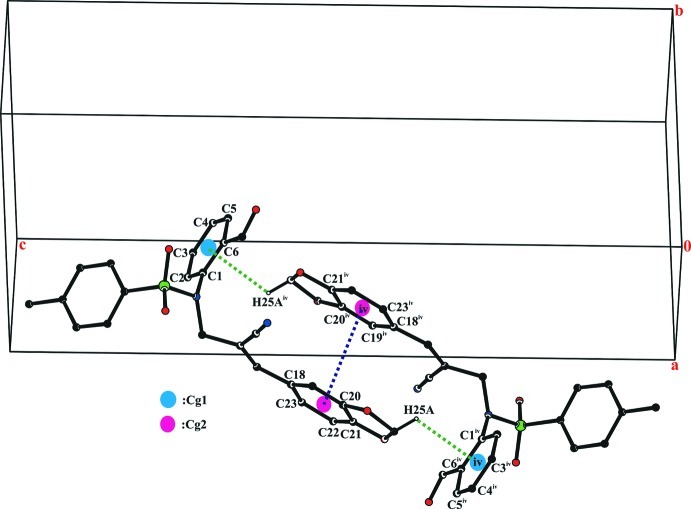
A view of the C—H···π and π—π interactions (dotted lines) in the crystal structure of the title compound. Cg1 and Cg2 denotes centroids of the C1–C6 benzene ring and C18–C23 benzene ring, respectively. [Symmetry codes: (iv)2-x,-y,1-z].

## supplementary crystallographic information

### Comment

Sulfonamide drugs are widely used for the treatment of certain infections caused by Gram-positive and Gram-negative microorganisms, some fungi, and certain protozoa (Korolkovas, 1988, Mandell & Sande, 1992). Benzodioxoles derivatives can be used as inhibitors of mono-oxygenase enzymes (Ullrich *et al.,* 2004), pesticides or pesticide intermediates (Gates & Gillon, 1974), herbicides (Arndt & Franke, 1977), antioxidants (Joshi *et al.,* 2005), antimicrobials (Jae *et al.,* 2001) and medicines (Leite *et al.,* 2004). In view of this biological importance, the crystal structure of the title compound has been determined and the results are presented here.

Fig. 1. shows a displacement ellipsoid plot of the title compound, with the atom numbering scheme. The S1 atom shows a distorted tetrahedral geometry, with O2—S1—O3[119.9 (1)°] and N1—S1—C8[107.0 (1)°] angles deviating from ideal tetrahedral values, are attributed to the Thrope-Ingold effect (Bassindale, 1984). The sum of bond angles around N1 (351°) indicates that N1 is in *sp*^2^ hybridization. The benzodioxole ring system is essentially planar [maximum deviation = 0.021 (2) Å for the O5 atom] and forms dihedral angles of 85.2 (1)° and 74.2 (1)°, respectively, with the formyl benzene and sulfonyl–bound benzene rings. The carbonitrile side chain (C16–C24–N2) is almost linear, with the angle around central carbon atom being 177.1 (2)°. The geometric parameters of the title molecule agree well with those reported for similar structures (Madhanraj *et al.,* 2011; Aziz-ur-Rehman *et al.,* 2010).

The molecular structure is stabilized by an C15—H15B···O3 intramolecular hydrogen bond, forming an S(5) ring motif (Bernstein *et al.,* 1995) (Table 1). The crystal packing is stabilized by intermolecular C—H···O hydrogen bonds. The formation of the framework can be explained in terms of two-one substructures. In the first substructure, atom C4 in the molecule at (*x, y, z*) acts as a hydrogen bond donor to atom O3 in the molecule at (*-1+x, y, z*) generating C(8) chains which are running along [100] (Fig. 2). In the second substructure, three molecules are linked by the combination of C15—H15B···O2 and C23—H23···O1 intermolecular hydrogen bonds generating *R_3_^3^*(19) ring motifs along [010] (Fig. 3). The crystal packing (Fig. 4) is further stabilized by weak C—H···π interactions between a dioxole H atom and the benzene ring (C1–C6) of a neighbouring molecule, with a C25—H25A···*Cg*1^iv^ distance of 3.446 (4) Å (Table 1; *Cg*1 is the centroid of the C1–C6 benzene ring, Symmetry code: iv = *2-x, -y, 1-z*). Additional stability arises from weak aromatic π—π interaction between the benzene rings of neighbouring molecules, with *Cg*2—*Cg*2^iv^ distance of 3.937 (3) Å (Fig. 4; *Cg*2 is the centroid of the C18—C23 benzene ring, symmetry code: iv = *2-x, -y, 1-z*).

### Experimental

To a stirred solution of *N*-(2-formylphenyl)-4-methylbenzenesulfonamide (0.275 g, 1 mmol) in acetonitrile (7 ml), potassium carbonate (0.35 g, 2.5 mmol) was added and stirred well for five minutes. To this solution, (*z*)-methyl 3-(benzo[d][1,3]dioxol-5-yl)-2- (bromomethyl)prop-2-enenitrile (0.299 g, 1 mmol) in acetonitrile (0.5 ml) was added and allowed to stir well for 6 h. After the completion of the reaction, the reaction mixture was poured into water and extracted using ethyl acetate. The organic layer thus obtained was concentrated under reduced pressure and the residual mass thus obtained was purified by column chromatography on silica gel (100–200 mesh) using ethylacetate and hexanes (1:9) as solvents. The pure title compound was obtained as a colourless solid (0.435 g, 94% yield). Recrystallization was carried out using ethylacetate as solvent.

### Refinement

All the H atoms were positioned geometrically with C–H = 0.93–0.97 Å and constrained to ride on their parent atom, with *U*_iso_(H) =1.5*U*_eq_ for methyl H atoms and 1.2*U*_eq_(C) for other H atoms.

### Crystal data

**Table:**

C_25_H_20_N_2_O_5_S	*F*(000) = 960
*M**_r_* = 460.49	*D*_x_ = 1.329 Mg m^−^^3^
Monoclinic, *P*2_1_/*n*	Mo *K*α radiation, λ = 0.71073 Å
Hall symbol: -P 2yn	Cell parameters from 6508 reflections
*a* = 8.921 (5) Å	θ = 2.2–29.6°
*b* = 10.235 (4) Å	µ = 0.18 mm^−^^1^
*c* = 25.256 (3) Å	*T* = 293 K
β = 93.380 (4)°	Block, colourless
*V* = 2302.0 (16) Å^3^	0.23 × 0.21 × 0.16 mm
*Z* = 4	

### Data collection

**Table:**

Bruker APEXII CCD diffractometer	6451 independent reflections
Radiation source: fine-focus sealed tube	3582 reflections with *I* > 2σ(*I*)
Graphite monochromator	*R*_int_ = 0.035
Detector resolution: 10.0 pixels mm^-1^	θ_max_ = 29.6°, θ_min_ = 2.2°
ω scans	*h* = −11→12
Absorption correction: multi-scan (*SADABS*; Sheldrick, 1996)	*k* = −12→14
*T*_min_ = 0.959, *T*_max_ = 0.972	*l* = −35→35
26810 measured reflections	

### Refinement

**Table:**

Refinement on *F*^2^	Primary atom site location: structure-invariant direct methods
Least-squares matrix: full	Secondary atom site location: difference Fourier map
*R*[*F*^2^ > 2σ(*F*^2^)] = 0.050	Hydrogen site location: inferred from neighbouring sites
*wR*(*F*^2^) = 0.148	H-atom parameters constrained
*S* = 1.01	*w* = 1/[σ^2^(*F*_o_^2^) + (0.061*P*)^2^ + 0.4367*P*] where *P* = (*F*_o_^2^ + 2*F*_c_^2^)/3
6451 reflections	(Δ/σ)_max_ = 0.001
299 parameters	Δρ_max_ = 0.24 e Å^−^^3^
0 restraints	Δρ_min_ = −0.28 e Å^−^^3^

### Special details

**Table:**

Geometry. All esds (except the esd in the dihedral angle between two l.s. planes) are estimated using the full covariance matrix. The cell esds are taken into account individually in the estimation of esds in distances, angles and torsion angles; correlations between esds in cell parameters are only used when they are defined by crystal symmetry. An approximate (isotropic) treatment of cell esds is used for estimating esds involving l.s. planes.
Refinement. Refinement of F^2^ against ALL reflections. The weighted R-factor wR and goodness of fit S are based on F^2^, conventional R-factors R are based on F, with F set to zero for negative F^2^. The threshold expression of F^2^ > 2sigma(F^2^) is used only for calculating R-factors(gt) etc. and is not relevant to the choice of reflections for refinement. R-factors based on F^2^ are statistically about twice as large as those based on F, and R- factors based on ALL data will be even larger.

### Fractional atomic coordinates and isotropic or equivalent isotropic displacement parameters (Å^2^)

**Table:**

	*x*	*y*	*z*	*U*_iso_*/*U*_eq_	
C1	0.88821 (17)	0.29000 (16)	0.70678 (7)	0.0460 (4)	
C2	0.7817 (2)	0.21821 (19)	0.73197 (8)	0.0583 (5)	
H2	0.8111	0.1480	0.7535	0.070*	
C3	0.6312 (2)	0.2512 (2)	0.72499 (9)	0.0708 (6)	
H3	0.5597	0.2020	0.7415	0.085*	
C4	0.5870 (2)	0.3551 (2)	0.69422 (9)	0.0720 (6)	
H4	0.4860	0.3776	0.6903	0.086*	
C5	0.6909 (2)	0.4257 (2)	0.66930 (9)	0.0690 (5)	
H5	0.6600	0.4964	0.6482	0.083*	
C6	0.8425 (2)	0.39395 (18)	0.67483 (8)	0.0571 (5)	
C7	0.9488 (3)	0.4706 (3)	0.64478 (12)	0.0993 (9)	
H7	1.0464	0.4397	0.6429	0.119*	
C8	1.0796 (2)	0.2967 (2)	0.82269 (8)	0.0615 (5)	
C9	1.1506 (3)	0.1941 (2)	0.84920 (10)	0.0804 (6)	
H9	1.2329	0.1535	0.8353	0.096*	
C10	1.0975 (4)	0.1524 (3)	0.89685 (12)	0.1011 (9)	
H10	1.1449	0.0831	0.9148	0.121*	
C11	0.9766 (4)	0.2113 (4)	0.91805 (12)	0.1072 (10)	
C12	0.9091 (3)	0.3127 (4)	0.89092 (13)	0.1068 (9)	
H12	0.8270	0.3534	0.9049	0.128*	
C13	0.9580 (3)	0.3563 (2)	0.84401 (10)	0.0836 (7)	
H13	0.9098	0.4257	0.8264	0.100*	
C14	0.9192 (5)	0.1625 (5)	0.96973 (14)	0.1774 (19)	
H14A	0.8719	0.2330	0.9874	0.266*	
H14B	1.0017	0.1297	0.9920	0.266*	
H14C	0.8476	0.0938	0.9625	0.266*	
C15	1.0965 (2)	0.12671 (18)	0.70817 (8)	0.0607 (5)	
H15A	1.0210	0.0672	0.7202	0.073*	
H15B	1.1883	0.1123	0.7299	0.073*	
C16	1.12431 (19)	0.09650 (18)	0.65121 (7)	0.0547 (4)	
C17	1.0763 (2)	−0.01502 (18)	0.62804 (8)	0.0570 (5)	
H17	1.0133	−0.0641	0.6483	0.068*	
C18	1.1044 (2)	−0.07351 (18)	0.57716 (7)	0.0570 (5)	
C19	1.2126 (3)	−0.0279 (2)	0.54337 (8)	0.0743 (6)	
H19	1.2694	0.0464	0.5516	0.089*	
C20	1.2309 (3)	−0.0963 (2)	0.49839 (9)	0.0778 (6)	
C21	1.1496 (3)	−0.2059 (2)	0.48521 (9)	0.0798 (6)	
C22	1.0463 (3)	−0.2538 (2)	0.51699 (10)	0.0911 (8)	
H22	0.9916	−0.3288	0.5082	0.109*	
C23	1.0258 (3)	−0.1855 (2)	0.56332 (9)	0.0741 (6)	
H23	0.9558	−0.2165	0.5861	0.089*	
C24	1.2139 (2)	0.1901 (2)	0.62545 (9)	0.0708 (6)	
C25	1.3046 (4)	−0.1707 (3)	0.42145 (11)	0.1120 (10)	
H25A	1.2715	−0.1314	0.3878	0.134*	
H25B	1.3966	−0.2186	0.4165	0.134*	
N1	1.04579 (15)	0.26209 (13)	0.71551 (6)	0.0523 (4)	
N2	1.2828 (3)	0.2688 (2)	0.60623 (9)	0.1089 (8)	
O1	0.9152 (3)	0.5697 (3)	0.62298 (14)	0.1952 (15)	
O2	1.08789 (15)	0.48120 (13)	0.75324 (7)	0.0788 (4)	
O3	1.29242 (13)	0.31885 (16)	0.75875 (7)	0.0844 (5)	
O4	1.1940 (3)	−0.2561 (2)	0.43819 (7)	0.1168 (7)	
O5	1.3313 (3)	−0.0722 (2)	0.46024 (7)	0.1254 (8)	
S1	1.13777 (5)	0.34993 (5)	0.76162 (2)	0.06199 (17)	

### Atomic displacement parameters (Å^2^)

**Table:**

	*U*^11^	*U*^22^	*U*^33^	*U*^12^	*U*^13^	*U*^23^
C1	0.0383 (8)	0.0409 (8)	0.0596 (10)	−0.0011 (7)	0.0085 (7)	−0.0013 (8)
C2	0.0554 (11)	0.0548 (11)	0.0657 (12)	−0.0087 (9)	0.0117 (9)	0.0054 (9)
C3	0.0455 (10)	0.0779 (14)	0.0907 (15)	−0.0188 (10)	0.0180 (10)	−0.0106 (12)
C4	0.0411 (10)	0.0852 (16)	0.0894 (15)	0.0038 (10)	0.0008 (10)	−0.0146 (13)
C5	0.0615 (12)	0.0646 (13)	0.0802 (14)	0.0145 (10)	−0.0026 (10)	0.0018 (11)
C6	0.0482 (10)	0.0492 (10)	0.0748 (12)	0.0024 (8)	0.0105 (9)	0.0069 (9)
C7	0.0858 (17)	0.0776 (16)	0.137 (2)	0.0021 (13)	0.0276 (16)	0.0496 (16)
C8	0.0434 (9)	0.0609 (11)	0.0794 (13)	−0.0038 (9)	−0.0038 (9)	−0.0227 (10)
C9	0.0644 (13)	0.0822 (16)	0.0931 (17)	0.0013 (12)	−0.0080 (12)	−0.0174 (14)
C10	0.107 (2)	0.105 (2)	0.0874 (19)	−0.0205 (18)	−0.0199 (17)	0.0057 (16)
C11	0.106 (2)	0.136 (3)	0.0801 (18)	−0.037 (2)	0.0101 (17)	−0.0326 (19)
C12	0.094 (2)	0.124 (2)	0.105 (2)	−0.0081 (19)	0.0311 (17)	−0.039 (2)
C13	0.0698 (14)	0.0851 (16)	0.0974 (18)	0.0046 (12)	0.0176 (13)	−0.0266 (14)
C14	0.203 (4)	0.244 (5)	0.088 (2)	−0.061 (4)	0.033 (2)	−0.002 (3)
C15	0.0602 (11)	0.0530 (10)	0.0693 (12)	0.0162 (9)	0.0083 (9)	−0.0018 (9)
C16	0.0461 (9)	0.0556 (11)	0.0625 (11)	0.0076 (8)	0.0058 (8)	0.0000 (9)
C17	0.0544 (10)	0.0524 (10)	0.0643 (11)	0.0041 (9)	0.0046 (9)	0.0066 (9)
C18	0.0611 (11)	0.0500 (10)	0.0593 (11)	0.0019 (9)	−0.0010 (9)	0.0032 (8)
C19	0.0934 (16)	0.0631 (12)	0.0672 (13)	−0.0196 (12)	0.0133 (11)	−0.0060 (10)
C20	0.1023 (17)	0.0726 (14)	0.0598 (13)	−0.0106 (13)	0.0150 (12)	−0.0013 (11)
C21	0.1077 (18)	0.0704 (14)	0.0607 (13)	−0.0056 (14)	0.0010 (12)	−0.0096 (11)
C22	0.110 (2)	0.0724 (15)	0.0909 (18)	−0.0265 (14)	0.0079 (15)	−0.0197 (13)
C23	0.0794 (14)	0.0626 (13)	0.0809 (15)	−0.0122 (11)	0.0103 (11)	−0.0005 (11)
C24	0.0687 (13)	0.0712 (13)	0.0741 (14)	−0.0141 (11)	0.0173 (11)	−0.0181 (11)
C25	0.156 (3)	0.115 (2)	0.0679 (16)	−0.012 (2)	0.0270 (17)	−0.0224 (16)
N1	0.0398 (7)	0.0451 (8)	0.0725 (10)	0.0052 (6)	0.0084 (7)	−0.0033 (7)
N2	0.1215 (18)	0.1053 (17)	0.1043 (16)	−0.0526 (15)	0.0437 (14)	−0.0253 (13)
O1	0.144 (2)	0.142 (2)	0.305 (4)	0.0164 (17)	0.054 (2)	0.157 (3)
O2	0.0565 (8)	0.0483 (8)	0.1309 (13)	−0.0080 (6)	0.0003 (8)	−0.0096 (8)
O3	0.0349 (7)	0.0990 (12)	0.1201 (13)	−0.0006 (7)	0.0105 (7)	−0.0133 (10)
O4	0.170 (2)	0.1052 (14)	0.0777 (12)	−0.0327 (14)	0.0295 (12)	−0.0314 (10)
O5	0.183 (2)	0.1159 (15)	0.0837 (12)	−0.0514 (15)	0.0614 (13)	−0.0252 (11)
S1	0.0339 (2)	0.0565 (3)	0.0959 (4)	−0.0033 (2)	0.0072 (2)	−0.0108 (3)

### Geometric parameters (Å, º)

**Table:**

C1—C6	1.382 (2)	C14—H14C	0.9600
C1—C2	1.385 (2)	C15—N1	1.473 (2)
C1—N1	1.439 (2)	C15—C16	1.506 (3)
C2—C3	1.386 (3)	C15—H15A	0.9700
C2—H2	0.9300	C15—H15B	0.9700
C3—C4	1.361 (3)	C16—C17	1.341 (3)
C3—H3	0.9300	C16—C24	1.429 (3)
C4—C5	1.359 (3)	C17—C18	1.453 (3)
C4—H4	0.9300	C17—H17	0.9300
C5—C6	1.390 (3)	C18—C23	1.378 (3)
C5—H5	0.9300	C18—C19	1.406 (3)
C6—C7	1.475 (3)	C19—C20	1.352 (3)
C7—O1	1.184 (3)	C19—H19	0.9300
C7—H7	0.9300	C20—C21	1.367 (3)
C8—C9	1.379 (3)	C20—O5	1.376 (3)
C8—C13	1.381 (3)	C21—C22	1.349 (3)
C8—S1	1.743 (2)	C21—O4	1.373 (3)
C9—C10	1.386 (4)	C22—C23	1.384 (3)
C9—H9	0.9300	C22—H22	0.9300
C10—C11	1.372 (4)	C23—H23	0.9300
C10—H10	0.9300	C24—N2	1.138 (3)
C11—C12	1.364 (4)	C25—O4	1.402 (3)
C11—C14	1.515 (5)	C25—O5	1.417 (3)
C12—C13	1.361 (4)	C25—H25A	0.9700
C12—H12	0.9300	C25—H25B	0.9700
C13—H13	0.9300	N1—S1	1.6508 (16)
C14—H14A	0.9600	O2—S1	1.4272 (15)
C14—H14B	0.9600	O3—S1	1.4219 (15)
			
C6—C1—C2	119.32 (16)	C16—C15—H15A	109.1
C6—C1—N1	119.61 (14)	N1—C15—H15B	109.1
C2—C1—N1	120.99 (16)	C16—C15—H15B	109.1
C1—C2—C3	119.88 (19)	H15A—C15—H15B	107.8
C1—C2—H2	120.1	C17—C16—C24	123.01 (18)
C3—C2—H2	120.1	C17—C16—C15	121.67 (17)
C4—C3—C2	120.55 (18)	C24—C16—C15	115.16 (17)
C4—C3—H3	119.7	C16—C17—C18	132.02 (18)
C2—C3—H3	119.7	C16—C17—H17	114.0
C5—C4—C3	119.87 (19)	C18—C17—H17	114.0
C5—C4—H4	120.1	C23—C18—C19	118.69 (19)
C3—C4—H4	120.1	C23—C18—C17	117.08 (18)
C4—C5—C6	121.0 (2)	C19—C18—C17	124.10 (18)
C4—C5—H5	119.5	C20—C19—C18	117.3 (2)
C6—C5—H5	119.5	C20—C19—H19	121.3
C1—C6—C5	119.36 (17)	C18—C19—H19	121.3
C1—C6—C7	122.22 (18)	C19—C20—C21	122.8 (2)
C5—C6—C7	118.39 (19)	C19—C20—O5	127.7 (2)
O1—C7—C6	122.8 (3)	C21—C20—O5	109.6 (2)
O1—C7—H7	118.6	C22—C21—C20	121.6 (2)
C6—C7—H7	118.6	C22—C21—O4	128.4 (2)
C9—C8—C13	119.7 (2)	C20—C21—O4	110.0 (2)
C9—C8—S1	121.03 (17)	C21—C22—C23	116.7 (2)
C13—C8—S1	119.24 (19)	C21—C22—H22	121.7
C8—C9—C10	119.1 (2)	C23—C22—H22	121.7
C8—C9—H9	120.5	C18—C23—C22	122.9 (2)
C10—C9—H9	120.5	C18—C23—H23	118.5
C11—C10—C9	121.3 (3)	C22—C23—H23	118.5
C11—C10—H10	119.3	N2—C24—C16	177.1 (2)
C9—C10—H10	119.3	O4—C25—O5	109.1 (2)
C12—C11—C10	118.2 (3)	O4—C25—H25A	109.9
C12—C11—C14	121.5 (4)	O5—C25—H25A	109.9
C10—C11—C14	120.3 (4)	O4—C25—H25B	109.9
C13—C12—C11	122.1 (3)	O5—C25—H25B	109.9
C13—C12—H12	118.9	H25A—C25—H25B	108.3
C11—C12—H12	118.9	C1—N1—C15	118.17 (14)
C12—C13—C8	119.6 (3)	C1—N1—S1	116.13 (11)
C12—C13—H13	120.2	C15—N1—S1	117.24 (12)
C8—C13—H13	120.2	C21—O4—C25	105.78 (19)
C11—C14—H14A	109.5	C20—O5—C25	105.5 (2)
C11—C14—H14B	109.5	O3—S1—O2	119.87 (9)
H14A—C14—H14B	109.5	O3—S1—N1	106.60 (9)
C11—C14—H14C	109.5	O2—S1—N1	105.69 (9)
H14A—C14—H14C	109.5	O3—S1—C8	108.38 (10)
H14B—C14—H14C	109.5	O2—S1—C8	108.60 (10)
N1—C15—C16	112.49 (15)	N1—S1—C8	107.01 (8)
N1—C15—H15A	109.1		
			
C6—C1—C2—C3	−0.1 (3)	O5—C20—C21—C22	177.8 (3)
N1—C1—C2—C3	176.75 (16)	C19—C20—C21—O4	−178.8 (2)
C1—C2—C3—C4	−1.1 (3)	O5—C20—C21—O4	−0.2 (3)
C2—C3—C4—C5	1.2 (3)	C20—C21—C22—C23	0.6 (4)
C3—C4—C5—C6	−0.2 (3)	O4—C21—C22—C23	178.3 (2)
C2—C1—C6—C5	1.1 (3)	C19—C18—C23—C22	−1.4 (3)
N1—C1—C6—C5	−175.84 (17)	C17—C18—C23—C22	−177.4 (2)
C2—C1—C6—C7	−177.0 (2)	C21—C22—C23—C18	0.5 (4)
N1—C1—C6—C7	6.1 (3)	C17—C16—C24—N2	152 (6)
C4—C5—C6—C1	−0.9 (3)	C15—C16—C24—N2	−32 (6)
C4—C5—C6—C7	177.2 (2)	C6—C1—N1—C15	−130.68 (18)
C1—C6—C7—O1	−169.7 (3)	C2—C1—N1—C15	52.5 (2)
C5—C6—C7—O1	12.2 (5)	C6—C1—N1—S1	82.06 (19)
C13—C8—C9—C10	0.3 (3)	C2—C1—N1—S1	−94.77 (18)
S1—C8—C9—C10	−177.96 (18)	C16—C15—N1—C1	85.0 (2)
C8—C9—C10—C11	−0.2 (4)	C16—C15—N1—S1	−128.14 (15)
C9—C10—C11—C12	0.1 (4)	C22—C21—O4—C25	−179.4 (3)
C9—C10—C11—C14	179.0 (3)	C20—C21—O4—C25	−1.6 (3)
C10—C11—C12—C13	0.0 (4)	O5—C25—O4—C21	2.8 (3)
C14—C11—C12—C13	−178.9 (3)	C19—C20—O5—C25	−179.6 (3)
C11—C12—C13—C8	0.1 (4)	C21—C20—O5—C25	1.9 (3)
C9—C8—C13—C12	−0.2 (3)	O4—C25—O5—C20	−2.9 (3)
S1—C8—C13—C12	178.05 (19)	C1—N1—S1—O3	−172.54 (13)
N1—C15—C16—C17	−135.66 (18)	C15—N1—S1—O3	39.89 (16)
N1—C15—C16—C24	48.7 (2)	C1—N1—S1—O2	−43.97 (14)
C24—C16—C17—C18	4.2 (3)	C15—N1—S1—O2	168.46 (13)
C15—C16—C17—C18	−171.09 (18)	C1—N1—S1—C8	71.65 (14)
C16—C17—C18—C23	−174.4 (2)	C15—N1—S1—C8	−75.91 (15)
C16—C17—C18—C19	9.9 (3)	C9—C8—S1—O3	−26.56 (19)
C23—C18—C19—C20	1.2 (3)	C13—C8—S1—O3	155.17 (17)
C17—C18—C19—C20	176.9 (2)	C9—C8—S1—O2	−158.29 (16)
C18—C19—C20—C21	−0.2 (4)	C13—C8—S1—O2	23.45 (19)
C18—C19—C20—O5	−178.5 (2)	C9—C8—S1—N1	88.05 (17)
C19—C20—C21—C22	−0.8 (4)	C13—C8—S1—N1	−90.22 (18)

### Hydrogen-bond geometry (Å, º)

Cg1 is the centroid of the C1–C6 benzene ring.

**Table:**

*D*—H···*A*	*D*—H	H···*A*	*D*···*A*	*D*—H···*A*
C15—H15*B*···O3	0.97	2.40	2.879 (3)	110
C15—H15*B*···O2^i^	0.97	2.42	3.282 (3)	148
C23—H23···O1^ii^	0.93	2.41	3.114 (3)	132
C4—H4···O3^iii^	0.93	2.59	3.195 (3)	124
C25—H25*A*···*Cg*1^iv^	0.97	2.96	3.446 (4)	112

Symmetry codes: (i) −*x*+5/2, *y*−1/2, −*z*+3/2; (ii) *x*, *y*−1, *z*; (iii) *x*−1, *y*, *z*; (iv) −*x*+2, −*y*, −*z*+1.

## References

[bb1] Arndt, F. & Franke, H. (1977). DE Patent No. 2624822.

[bb2] Aziz-ur-Rehman, Tanveer, W., Akkurt, M., Sattar, A., Abbasi, M. A. & Khan, I. U. (2010). *Acta Cryst.* E**66**, o2980. 10.1107/S1600536810043369 PMC3009330 21589146

[bb3] Bassindale, A. (1984). *The Third Dimension in Organic Chemistry.* New York: John Wiley and Sons.

[bb4] Bernstein, J., Davis, R. E., Shimoni, L. & Chang, N.-L. (1995). *Angew. Chem. Int. Ed. Engl.* **34**, 1555–1573.

[bb5] Bruker (2004). *APEX2, *SAINT** and *XPREP* Bruker AXS Inc., Madison, Wisconsin, USA.

[bb6] Farrugia, L. J. (1997). *J. Appl. Cryst.* **30**, 565.

[bb7] Gates, P. S. & Gillon, J. (1974). US Patent No. 3 736 338.

[bb8] Jae, H.-S., Win, M., von Geldern, T. W., Sorensen, B. K., Chiou, W. J., Nguyen, B., Marsh, K. C. & Opgenorth, T. J. (2001). *J. Med. Chem.* **44**, 3978–3984. 10.1021/jm010237l 11689084

[bb9] Joshi, R., Kumar, M. S., Satyamoorthy, K., Unnikrisnan, M. K. & Mukherjee, T. (2005). *J. Agric. Food Chem.* **53**, 2696–2703. 10.1021/jf0489769 15796613

[bb10] Korolkovas, A. (1988). In *Essentials of Medicinal Chemistry*, 2nd ed., pp. 699–716. New York: Wiley.

[bb11] Leite, A. C. L., Peixoto da Silva, K., de Souza, I. A., Magali de Araujo, J. & Brondani, D. J. (2004). *Eur. J. Med. Chem.* **39**, 1059–1065. 10.1016/j.ejmech.2004.09.007 15571867

[bb12] Madhanraj, R., Murugavel, S., Kannan, D. & Bakthadoss, M. (2011). *Acta Cryst.* E**67**, o3511. 10.1107/S1600536811050756 PMC3239132 22199980

[bb13] Mandell, G. L. & Sande, M. A. (1992). *In Goodman and Gilman, The Pharmacological Basis of Therapeutics 2*, edited by A. Gilman, T. W. Rall, A. S. Nies & P. Taylor, 8th ed., pp. 1047–1057. Singapore: McGraw-Hill.

[bb14] Sheldrick, G. M. (1996). *SADABS* University of Göttingen, Germany.

[bb15] Sheldrick, G. M. (2008). *Acta Cryst.* A**64**, 112–122. 10.1107/S0108767307043930 18156677

[bb16] Spek, A. L. (2009). *Acta Cryst.* D**65**, 148–155. 10.1107/S090744490804362X PMC2631630 19171970

[bb17] Ullrich, T., Baumann, K., Welzenbach, K., Schmutz, S., Camenish, G., Meingassner, J. G. & Weitz-Schmidt, G. (2004). *Bioorg. Med. Chem. Lett.* **14**, 2483–2487. 10.1016/j.bmcl.2004.03.006 15109637

